# Concurrent Measurement of Dynamic Changes in Viral Load, Serum Enzymes, T Cell Subsets, and Cytokines in Patients with Severe Fever with Thrombocytopenia Syndrome

**DOI:** 10.1371/journal.pone.0091679

**Published:** 2014-03-21

**Authors:** Jun Li, Yaping Han, Yiping Xing, Shuang Li, Lianhua Kong, Yongxiang Zhang, Lili Zhang, Ning Liu, Qian Wang, Shixia Wang, Shan Lu, Zuhu Huang

**Affiliations:** 1 Department of Infectious Diseases, The First Affiliated Hospital, Nanjing Medical University, Nanjing, China; 2 China-US Vaccine Research Center, The First Affiliated Hospital, Nanjing Medical University, Nanjing, China; 3 Laboratory of Nucleic Acid Vaccines, Department of Medicine, University of Massachusetts Medical School, Worcester, Massachusetts, United States of America; University of Illinois at Chicago, United States of America

## Abstract

Severe fever with thrombocytopenia syndrome (SFTS) is an emerging infection caused by a novel Bunyavirus. Analysis on the dynamic changes of clinical, laboratory, and immunological abnormalities associated with SFTS in a concurrent study is lacking. Thirty-three SFTS patients were admitted to Jiangsu People's Hospital, Nanjing, China, and diagnosis was made based on the clinical symptoms and positive viral RNA detected by RT-PCR. Four patients deceased and twenty-nine survived. Blood samples were collected every other day between Day 5 and Day 15 from the onset of fever. Samples from healthy volunteers were used as normal controls. Peak viral RNA load, serum enzymes, IL-6, and IL-10 were significantly higher in deceased patients compared to survivors. Viral load, serum enzymes, and cytokines declined in survivors within 2 weeks from onset of fever. CD69+ T cells were elevated early after infection while HLA-DR+ and CTLA4+ T cells were elevated during the recovery phase of those who survived. High level SFTSV viral load was concurrently observed with reduced PLT, elevated serum enzymes, elevated pro-inflammatory and anti-inflammatory cytokines, and activation of CD69+ T cells. The degree and pattern of changes in these parameters may indicate the clinical outcome in SFTSV-infected patients.

## Introduction

Severe fever with thrombocytopenia syndrome (SFTS) is an emerging infectious disease recently discovered in China [1]–[5]. The causative agent of this illness, severe fever with thrombocytopenia syndrome virus (SFTSV), was identified as a novel tick-borne Bunynavirus in genus Phlebovirus; it is also named Huaiyangshan virus, since the original cases were identified in the Huaiyangshan Mountains in 2009 [1], [6], [7]. It was reported that SFTSV can be transmitted in several ways: 1) patients can be infected through a tick bite; the virus has been detected in Haemaphysalis longicornis ticks [1], [7] and/or 2) through person-to-person transmission via contact with blood from patients with SFTS [8]. Sporadic and clustered SFTS endemics have been documented in at least six provinces in Northeastern, Eastern, and Central China since 2009 [1], .

The typical clinical presentation of SFTS is acute fever and thrombocytopenia (platelet count less than 100,000/ml), in addition to other non-specific features including muscle pain, severe malaise, nausea, vomiting, and diarrhea [1], [3], [6]–[8]. A high mortality rate (ranging from 12%–30%) has been reported for SFTSV-infected patients [1], [6], . The exact mechanism of SFTSV pathogenesis remains unclear but it is generally suspected that immunopathology plays a key role [10], [11]. As with other viral infections, immune activation and exaggerated cytokine production in the form of cytokine storm can potentially drive the SFTS disease process. Several studies reported that SFTSV infection could lead to elevated levels of serum cytokines, which might contribute to disease severity and clinical outcome [6], [12], [13]. In separate reports it was shown that the viral infection induced CD3+CD8+ and CD3+CD4+ T cell population changes [11], [14]. However, almost all of the previous reports showed data collected at one or two time points of the disease process rather than determining dynamic changes in key lab test results and immunological markers incurred during the critical clinical period shortly after infection.

In the current report, changes in SFTSV viral load, platelets and white blood cell counts, levels of key serum enzymes, cytokine profile and changes in two important T subset populations, were measured every other day during the first 10–15 days of hospitalization for four deceased patients and twenty-nine survivors diagnosed with SFTSV infection. Information learned from the current study provide a better understanding on the relationship between clinical disease progression and key clinical lab and immunological parameters. Such information is also useful to guide a more in-depth investigation on the mechanisms of SFTSV pathogenesis.

## Materials and Methods

### Participants

Between May 2011 and July 2013, thirty-three patients (16 males and 17 females) (Table 1), with confirmed SFTSV infection based on diagnostic guidelines from the Chinese Ministry of Health [15], were admitted to the Department of Infectious Diseases, First Affiliated Hospital of Nanjing Medical University, Nanjing, China. These patients were from the rural areas of three Eastern China provinces (17 patients from Anhui, 16 patients from Jiangsu, and 1 from Shandong). There was no geographical connection between the four patients who died (2 from Anhui and 2 from Jiangsu). In addition to the SFTSV-infected patients, thirty-two healthy volunteers (21 males and 11 females) were also enrolled in this study (Table 1) to serve as normal controls. Written informed consent was obtained from all participants and the study was approved by the Institutional Review Board at First Affiliated Hospital of Nanjing Medical University.

**Table 1:** Study cohorts.

Groups	No. of Subjects	Age range (average)	Gender (M/F)	Day of admission (range, median)*
Survivors	21	33–78 (58)	11/10	3–10 (5)
Deceased patients	3	58–75 (70)	2/1	7 (7)
Healthy Controls	32	18–60 (42)	21/11	NA

* Days after symptom onset.

### Sample collection and processing

All SFTSV-infected patients received standard antiviral and standard support treatments after admission to the hospital based on SFTS treatment guidelines from the Chinese Ministry of Health. Individual identification codes were given to each participant and blood samples were collected under identification codes. For SFTSV-infected patients, samples were collected during hospitalization at Days 5, 7, 9, 11, 13, and 15 after onset of fever while blood samples from healthy donors were collected at a single time point at the time of enrollment.

### Lab tests and assays

Blood cell and platelet counts were measured by Japanese SYSMEX XE2100 fully-automatic blood cell analyzer. Japanese Olympus AU5400 fully-automatic biochemical detector was used to determine levels of alanine transaminase (ALT), aspartate transaminase (AST), lactic dehydrogenase (LDH), and creatine kinase (CK).

The TaqMan real-time fluorescent quantitative PCR method was used to detect SFTSV viral load. The virus RNA was extracted from the plasma samples using a RNA extraction kit and RT-PCR amplification was conducted immediately on the fluorescent quantitative PCR detector (ABI StepOne Plus). SFTSV RNA was detected with the specific primers targeting the highly conserved region of SFTSV L, M, and S genes [1], [16]. The RT-PCR reaction system was in a volume of 25 µl; reaction conditions were 50°C for 15 min with 1 cycle; 95°C for 15 min with 1 cycle; 94°C for 15 sec, 55°C for 45 sec, and 55°C with 45 cycles. Fluorescent signals were collected by FAM channel.

Lymphocyte subpopulations and T cell activation profiles in patients' PBMC were analyzed by flow cytometry with florescent-conjugated T cell surface markers: florescent-conjugated mouse anti-human CD3-PerCP Cy5.5, CTLA-4-PE, CD4-FITC, CD28-PE, CD8-PE, CD69-FITC, CD25-PE, CD56-PE, HLA-DR-FITC, and isotype controls: mouse IgG1-PE and mouse IgG1-FITC (BD Bioscience, CA, USA). Cell populations were analyzed with FACS Calibur Flow Cytometer and CellQuest software (BD BioScience).

Serum cytokines were detected using Human Th1/Th2/Th17 Cytometric Bead Array (CBA) Kit (BD BioScience) according to the manufacturer's instructions. The flow cytometer (FACS Caliber) was used to detect IL-2, IL-4, IL-6, IL-10, TNF –α, IFN-γ, and IL-17A levels in the serum. The detection sensitivity of each cytokine was 2.5 pg/ml.

### Statistical analysis

The results were analyzed using statistical software SPSS17.0 for Windows (SPPS, an IBM Company). Mann-Whitney U non-parametric test, Student's t-test, or Rank-sum test, where appropriate, were used to compare the mean value of any two groups (deceased patients vs. survivors, or infected groups vs. normal control group), with *p<0.05* considered statistically significant.

## Results

During the period from May 2011 and July 2013, 33 patients with laboratory-confirmed SFTSV infection were admitted to Jiangsu People's Hospital, Nanjing, China. These patients share the similar clinic manifestations of SFTS, including acute fever (>38°C), weakness, anorexia, nausea and vomiting, as well as disturbance of consciousness, ecchymosis, and gastrointestinal hemorrhage for those in critical condition (Table 1), as well as the hallmark thrombocytopenia (platelet count <4×10^9^/L). At admission, all patients enrolled in this study had confirmed positive SFTSV in blood samples determined by reverse-transcriptase polymerase chain reaction (RT-PCR) with designated primers for SFTSV L, M, S genes. During hospitalization, all patients received similar treatment based on guidelines from the Chinese Ministry of Health [15]. Four died within 1–3 days after admission (7–9 days after onset of fever) and 29 patients recovered from the illness. The length between symptom onset to admission did not contribute to the mortality as those deceased were admitted on Day 7 of symptom onset while the median time of admission for those who survived was 6 days (range 3–10 days) (Table 1). There was no gender bias for the deceased (2 males/2 females) and survivors (14 males/16 females) patients. On the other hand, the median age of the deceased patients (70 years) was notably older than the median age of survivors (59 years), but not statistically significant due to a small sample size (Table 1).

**Figure 1:**
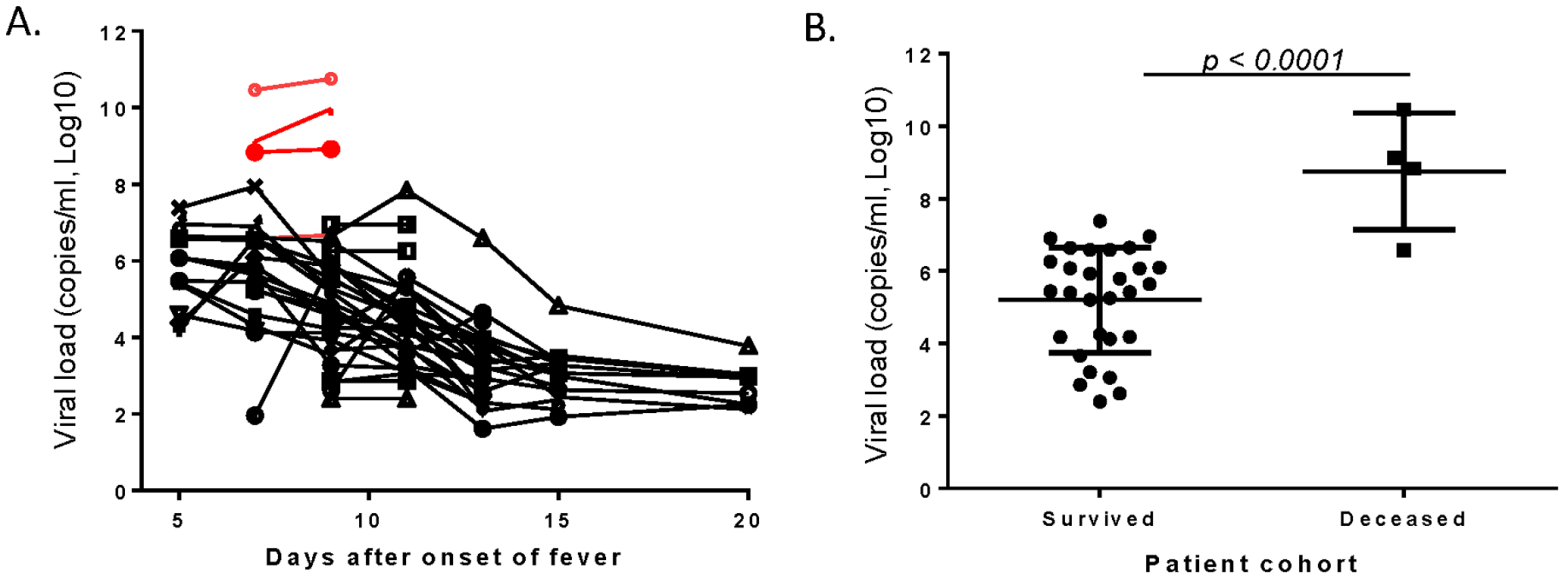
Severe fever with thrombocytopenia syndrome virus (SFTSV) RNA loads in blood. (A) The kinetics of viral RNA levels of individual patients between 5–15 days after onset of fever during hospitalization. Each curve represents one patient. The red curves indicate the deceased patients while the black curves represent the survivors. (B) The viral loads of the deceased patients and survivors as indicated on Day 9 after onset of fever. Each dot represents one patient. Statistical significance (*p<0.0001*) of viral loads between deceased patients and survivors is indicated.

While all patients had easily detectable SFTSV in their blood, SFTSV viral load among survivors peaked shortly after admission (within 7–11 days of symptom onset) and then dropped rapidly during the remaining period of the study (Fig. 1A). In contrast, three out of four deceased patients who were admitted on Day 7 of disease onset died within three days of admission with much higher levels of viral load while one of the deceased patients had similar levels of viral load to those who survived (Fig. 1A). When the viral loads on Day 9 for both groups were compared, the mean log viral load for the deceased patients (8.85, range 6.67–9.76) was about 1000 fold higher than the mean log viral load in the survivors (5.92, range 2.66–7.94) (*p<0.0001*) (Fig. 1B).

**Figure 2:**
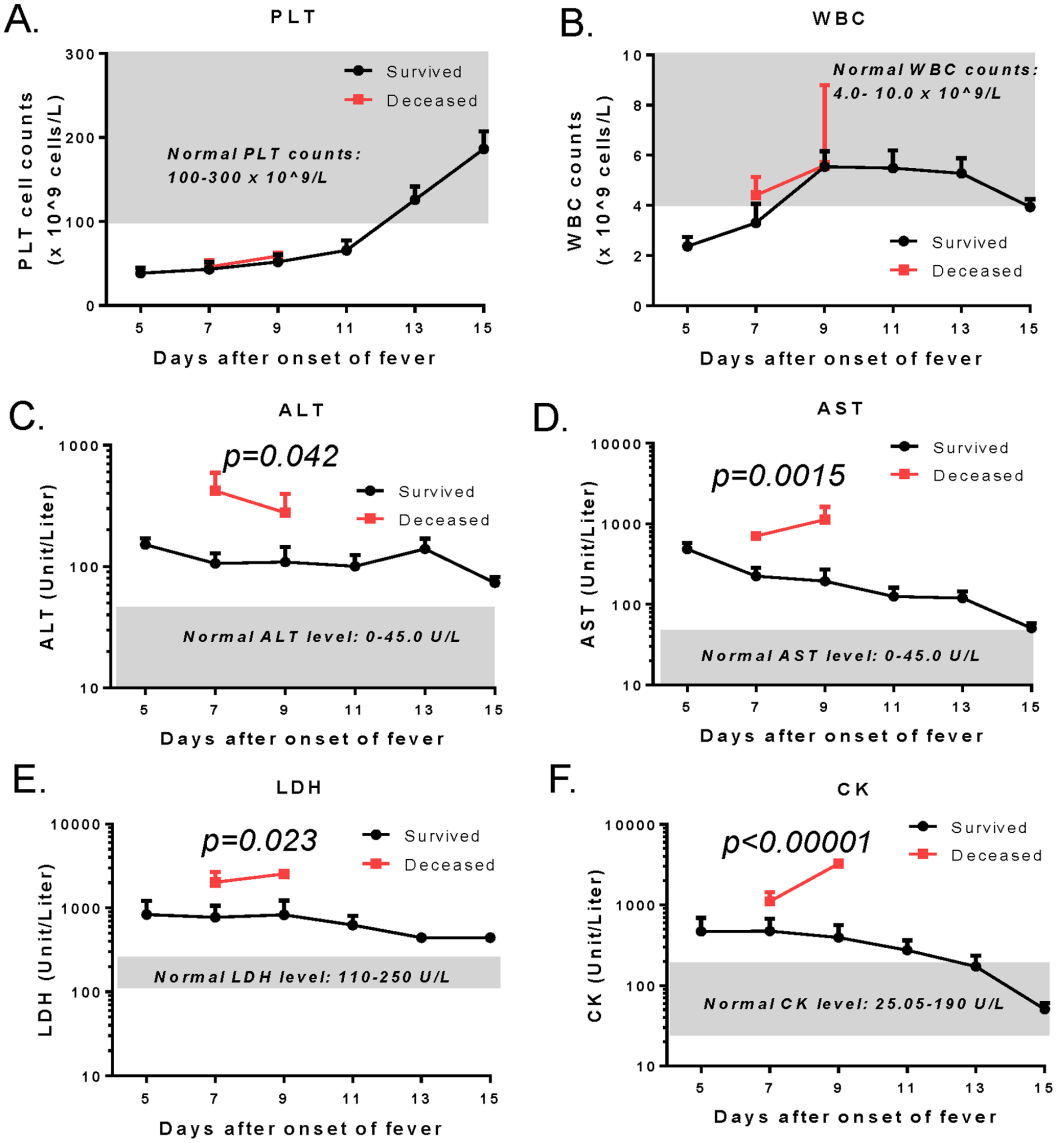
The kinetics of platelet (PLT) (A) and white blood cell (WBC) (B) counts, serum alanine transaminase (ALT) (C), aspartate transaminase (AST) (D), lactic dehydrogenase (LDH) (E), and creatine kinase (CK) (F) in SFTSV-infected patients. The red and black curves represent the deceased patients or the survivors, respectively, as the mean cell counts with standard error (A and B) or mean enzyme units/liter of blood with standard error (C–F) at each time point during hospitalization from Day 5 to Day 15 after onset of fever. The grey area in each graph indicates the normal range of related lab tests. Statistical significance (*p<0.05*) was indicated for each enzyme between deceased patients and survivors on Day 9 from onset of fever (C–F).

As indicated by the namesake of this disease, platelet (PLT) counts in all SFTSV-infected patients were low when the diagnosis was made, as evidenced by levels half of the normal range (100–300×10^9^/L). The level of drop in PTL was similar between deceased patients and survivors (Fig. 2A). After patients were admitted to hospital, their PLT levels improved steadily, partially due to treatment. PLT levels returned to a normal range on Day 13 among the survivors while those who died did so before Day 9 of disease onset with low PLT levels.

Parallel to PLT, white blood cell (WBC) counts were also below the normal range (4.0–9.0×10^9^/L) between 5–6 days after onset of fever but returned to normal around Day 9, earlier than PLT (Fig. 2B). Interestingly, those who died had WBC counts within normal range, albeit at the lower end of the normal range. It is likely that these patients were admitted on Day 7, slightly later than many of the other patients, and their WBC went up further after admission.

SFTSV-infected patients had acute tissue damage as indicated by multiple elevated key serum enzymes (Fig. 2C–F). ALT, AST, CK, and LDH were all highly elevated in both deceased patients and survivors; levels in the deceased patients were significantly higher than those in the survivors (Fig. 2C–F, Table 2). Our analysis further demonstrated that peak elevations were at the time of admission, about 5 days after the onset of symptoms. AST, CK, and LDH levels gradually declined among the survivors while ALT levels remained elevated for a longer time. All four patients died before Day 9 from symptom onset while all these biomarkers were highly elevated.

**Table 2:** Critical parameters that may be associated the severity of SFTS.

Parameters	Survivors	Fatal cases	
	Mean±SE	Mean±SE	*p value*
*Clinical Parameters*			
ALT (Units/L)	135.46±42.48	161.49±118.75	*>0.05*
AST (Units/L)	233.46±89.97	1028.26±688.01	*<0.01*
LDH (Units/L)	827.79±398.59	2550.18±120.02	*<0.001*
CK (Units/L)	419.13±209.92	3264.16±332.01	*<0.001*
*Serum Cytokines*			
IL-6 (pg/ml)	2.85±6.06	1268.88±703.47	*<0.0001*
IL-10 (pg/ml)	2.97±3.53	48.089±11.42	*<0.001*

**Figure 3:**
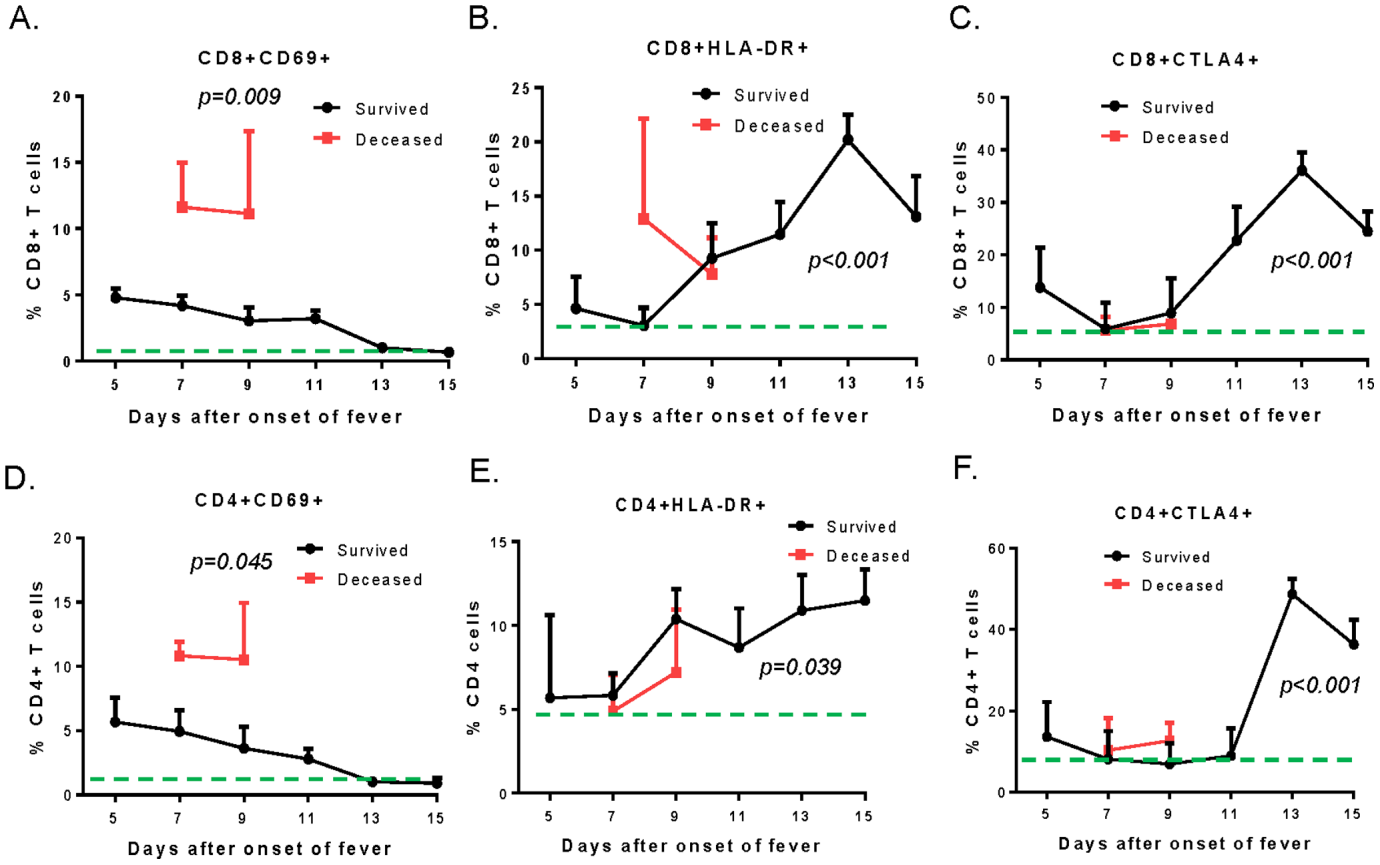
Dynamic changes in T cell subsets in SFTSV-infected patients. The red and black curves represent the deceased patient group and the survivors, respectively. The values are presented as mean percentages with standard error of each T cell subset at each time point during hospitalization from Day 5 to Day15 days from onset of fever. The green dashed line in each graph indicates the mean percentage in the normal control group. Statistical significance (*p<0.05*) was indicated in graphs A and C for the difference of individual T cell subsets between deceased patients and survivors on Day 9 from onset of fever, and in graphs B, D, E and F for the difference of individual T cell subsets between survivors and the normal control group on Day 13 from onset of fever.

Because it is suspected that virus-induced immunopathology may be involved in the tissue damage observed in both clinical presentation and abnormal lab tests, changes in important T cell subsets in SFTSV-infected patients were measured. Both CD8+/CD69+ and CD4+/CD69+ T cells were significantly elevated early in infection (Fig. 3A and 3B). At Day 9 after disease onset, mean percentages of CD4+/CD69+ T cells were 3.62% in survivors and 10.52% in later deceased patients while the normal control group was 1.12%. CD8+/CD69+ T cells were 3.02% in survivors and 11.15% in later deceased patients while the normal control group was 0.51%. Elevations in these T cells in later deceased patients were significantly higher than the elevation observed in survivors (p = 0.045 for CD4+/CD69+ T cells and p = 0.009 for CD8+/CD69+ T cells). These T cells gradually returned to normal levels in survivors around 2 weeks after disease onset.

HLA-DR+ T cells were also measured (Fig. 3C and 3D). The deceased patients had an early elevation of CD8+/HLA-DR+ T cells while these cells went up later among survivors, reaching peak levels at Day 13 after disease onset before coming down, although remaining higher than the normal control group (p<0.001). In contrast, CD4+/HLA-DR+ CD4 T cells went up at the same time for both survivors and deceased patients, and the levels continued to go up through the entire study period for survivors and were also much higher than observed for the normal control group (p = 0.039).

CTLA4+ T cells showed a different pattern. Both CD8+/CTLA4+ and CD4+/CTLA4+T cells went up around Day 11–13 from disease onset (Fig. 3E and 3F) and continued to go higher than observed in the normal control group during the remaining study period (p<0.001). For deceased patients, they died around Day 9 so CTLA4+ T cell levels were not significantly elevated. CD28+ T cells were also measured but no significant differences were observed between SFTSV-infected patients and healthy donors (data not shown).

**Figure 4:**
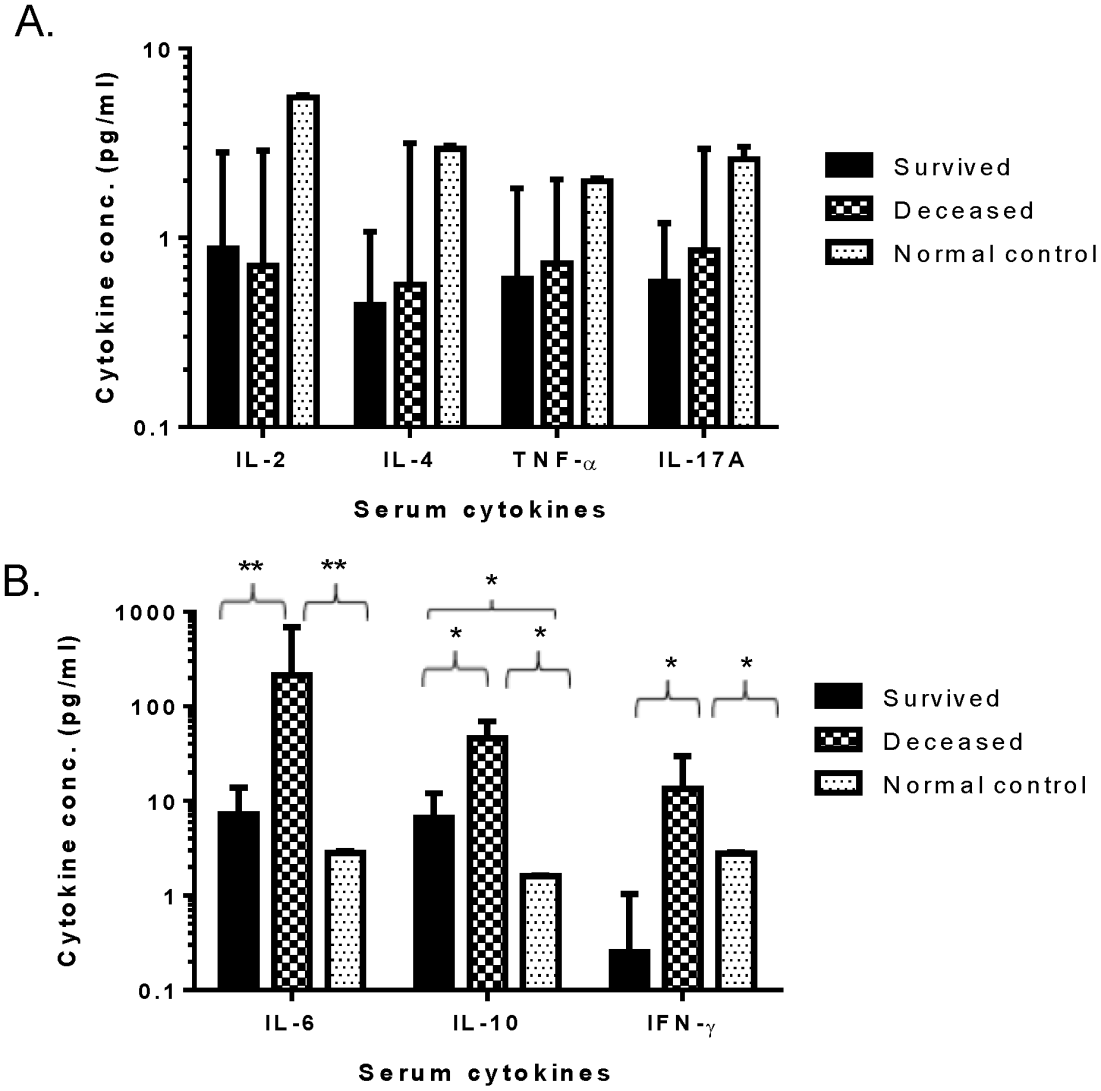
Serum cytokine profiles in SFTSV-infected patients on Day 7 after onset of fever. (A) Cytokines (IL-2, IL-4, TNF-α, and IL-17) that were not significantly different between SFTSV-infected patients and normal control group. (B) Cytokines (IL-6, IL-10, and IFN-γ) that were significantly different between SFTSV-infected patients and normal healthy donors. For each cytokine, * and ** indicate the statistical significance at p<0.05 and p<0.001, respectively.

**Figure 5:**
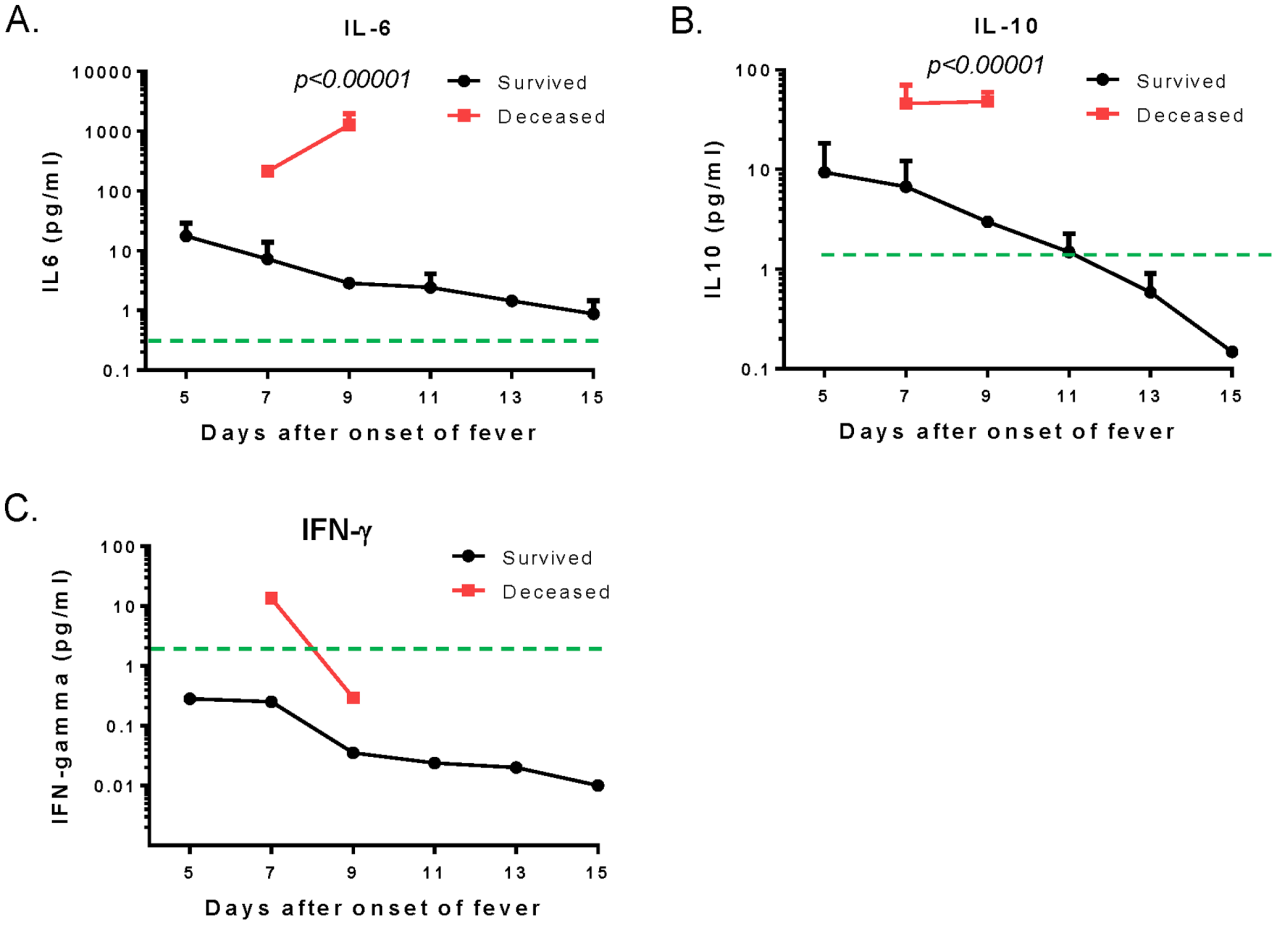
The kinetics of serum IL-6 (A), IL-10 (B), and IFN-γ (C) in SFTSV-infected patients during hospitalization between Day 5 and Day 15 from onset of fever. In all three graphs, the red and black curves represent the deceased patient group and the survivors, respectively. The values were presented as mean cytokine concentrations with standard errors at each time point. The green dashed line in each graph indicates mean cytokine concentrations in the normal control group. Statistical significance (*p<0.05*) was indicated between deceased patients and survivors on Day 9 from onset of fever.

Specific cytokines were measured further to identify those that may be involved in the pathogenesis of SFTS. Among the seven cytokines measured in this study, there were no significant differences for IL-2, IL-4, TNF-α, or IL-17A in serum samples between SFTSV-infected patients (both deceased and survivors) and the normal control group (Fig. 4A). However, significant elevations of serum IL-6, IL-10, and IFN-γ were discovered in deceased patients, not only compared to the normal control group but also to survivors (Fig. 4B). For survivors, both IL-6 and IL-10 were elevated but significantly only compared to the normal control group for IL-10 (Fig. 4B, Table 2). IFN-γ was actually lower in the survivors compared to the normal control group (Fig. 4B). When the kinetics of these three elevated cytokines were monitored, IL-6 and IL-10 levels showed an upward trend for the deceased patients; however, in survivors, levels slowly returned to normal levels (Fig. 5). IFN-γ went down for both deceased patients and survivors.

## Discussion

SFTS, which has a 12–30% mortality rate, is one of the most severe emerging human infectious diseases in China after severe acute respiratory syndrome (SARS) and avian influenza in the new millennium. While previous studies have documented the overall levels of viral load, changes in key lab results, and certain immunological biomarkers in SFTS patients at a few select time points, there is no report on the concurrent measurement of key abnormal findings over the critical acute phase of SFTS after infection, and no further analysis to relate these dynamic changes to the severity and course of the disease.

Although thrombocytopenia is a key abnormal finding in SFTS, its levels in both deceased patients and survivors were very similar in our analysis, and thus, may not be directly related to disease severity and death. In contrast, the level of SFTSV viremia is correlated to disease severity with significantly higher levels in the deceased patients than in the survivors when measured on Days 7–9 after onset of fever. Viral loads gradually dropped among survivors during the recovery phase. The finding is consistent with other reports that the SFTSV viral load is related to disease severity [6].

SFTSV infection causes tissue injury and organ damage, which can lead to elevated serum enzymatic markers and death. In all SFTSV-infected patients, ALT, AST, LDH, and CK were all elevated above the normal range especially during the acute phase of the disease, and their peak levels in deceased patients were significantly higher than in survivors, which is similar to other reports [6], [14] [10]. On the other hand, our kinetics analysis discovered that ALT was the last one to come down while AST, LDH and CK started to decrease among survivors very early after their admission to the hospital. These lab tests may serve great value for the clinical prognosis of SFTSV-infected patients.

The human immune system responds powerfully to a major viral infection such as SFTSV. Previous studies found that total CD3+ T cells and total CD4+ T cells were decreased in SFTSV-infected patients, more so during acute phase infection, in severe cases, and in those who eventually die from the disease [11]. At the same time, there is no significant change in CD8+ T cells [11], [14]. Because the measurement of serum cytokines in SFTSV-infected patients in previous studies actually showed increased levels consistent with a cytokine storm as expected in a typical major viral infection, the above data of total T cells was surprising. In the current study, change within subpopulations of T cells was investigated and profound T cell activation was discovered.

Three subsets of T cells (CD69+, HLA-DR+ and CTLA4+) were found elevated in SFTSV-infected patients but their changes happened at different phases of progression of the disease. CD69 molecule is a human transmembrane C-Type lectin protein and T cell early activation marker; CD69+ T cells (both CD8+ and CD4+) were elevated during the acute phase of SFTSV infection with significant activation in the deceased patients compared to the survivors at time points of the peak viremia. This T cell subpopulation in survivors gradually decreased to normal levels and was comparable to healthy donors in 2 weeks.

In contrast, HLA-DR+ (a MHC class II cell surface receptor) and CTLA4+ (a member of the immunoglobulin superfamily on the surface of helper T cells) T cells (both CD4+ and CD8+) were elevated during the recovery phase (after the peak viremia) in the survivors. These cells were low and only measured during the acute phase of infection for the deceased patients; however, HLA-DR+/CD8+ T cells were significantly elevated.

An increase in HLA-DR+ T cells is often in response to antigen binding and signaling. These cells bind to antigens on the surface of B cells, stimulating B cell proliferation. CTLA4 is a T-cell co-stimulatory molecule on antigen-presenting cells and transmits an inhibitory signal to T cells. HLA-DR and CTLA4 may serve as upregulation markers in the recovery phase and may have a role in checking and re-balancing the lymphocytes once the viral infection is under control. Taken together, our data imply that an increase inCD69+ T cells may be related to pathogenesis while a late increase in HLA-DR+ and CTLA4+ T cells may indicate the induction of protective immune responses.

As reported in SARS [17] and pandemic influenza cases [18], [19], patients infected with SFTSV also mounted an exaggerated immune response; furthermore, cytokine storm (hypercytokinemia) was responsible for significant damage to body tissues and organs, potentially resulting in death. In our study, both pro-inflammatory cytokines (such as IL-6 and IFN-γ) and anti-inflammatory cytokines (such as IL-10) were elevated in SFTSV-infected patients and levels were dramatically higher in the deceased patients, which is similar to reports by other groups [6], [10]. More significantly, IL-6 and IL-10 were persistently elevated in the deceased patients while levels trended down in survivors, indicating the possibility of using them as biomarkers to monitor the progression of the disease. Levels of IL-2, IL-4, TNF-α, and IL-17A were similar to normal healthy donors; this finding is somewhat different from previous reports showing that TNF-α was significantly elevated in SFTS-infected patients, especially deceased patients [6], [10]. It is not clear what may have contributed to this difference but timing of sample collection and different assay kits are potential factors influencing the final results of measurement.

In conclusion, the current study identified concurrent patterns of dynamic changes for viral load, PLT, key serum enzymes, major T cell subsets and unique cytokines in SFTS patients, further extending our understanding of this high risk, emerging viral infection, which was discovered only a few years ago. Findings on the involvement of CD69+ as well as HLA-DR+ and CTLA4+ T cells in SFTSV infection will lead more targeted research on the mechanism of pathogenesis in the coming future.

## References

[1] Yu XJ, Liang MF, Zhang SY, Liu Y, Li JD, et al (2011) Fever with thrombocytopenia associated with a novel bunyavirus in China. N Engl J Med 364: 1523–1532. 21410387 10.1056/NEJMoa1010095 PMC3113718

[2] Li S, Xue C, Fu Y, Wang J, Ding X, et al (2011) Sporadic case infected by severe fever with thrombocytopenia syndrome bunyavirus in a non-epidemic region of China. Biosci Trends 5: 273–276. 22281541 10.5582/bst.2011.v5.6.273

[3] Xu B, Liu L, Huang X, Ma H, Zhang Y, et al (2011) Metagenomic analysis of fever, thrombocytopenia and leukopenia syndrome (FTLS) in Henan Province, China: discovery of a new bunyavirus. PLoS Pathog 7: e1002369. 22114553 10.1371/journal.ppat.1002369 PMC3219706

[4] Bao CJ, Guo XL, Qi X, Hu JL, Zhou MH, et al (2011) A family cluster of infections by a newly recognized bunyavirus in eastern China, 2007: further evidence of person-to-person transmission. Clin Infect Dis 53: 1208–1214. 22028437 10.1093/cid/cir732

[5] Li D (2013) A highly pathogenic new bunyavirus emerged in China. Emerg Microbes Infect 2 doi:10.1038/emi.2013.1031 10.1038/emi.2013.1 PMC3630492 26038435

[6] Zhang YZ, He YW, Dai YA, Xiong Y, Zheng H, et al (2012) Hemorrhagic fever caused by a novel Bunyavirus in China: pathogenesis and correlates of fatal outcome. Clin Infect Dis 54: 527–533. 22144540 10.1093/cid/cir804

[7] Zhang YZ, Zhou DJ, Xiong Y, Chen XP, He YW, et al (2011) Hemorrhagic fever caused by a novel tick-borne Bunyavirus in Huaiyangshan, China. Zhonghua Liu Xing Bing Xue Za Zhi 32: 209–220. 21457654

[8] Liu Y, Li Q, Hu W, Wu J, Wang Y, et al (2012) Person-to-person transmission of severe fever with thrombocytopenia syndrome virus. Vector Borne Zoonotic Dis 12: 156–160. 21955213 10.1089/vbz.2011.0758

[9] Zhang X, Liu Y, Zhao L, Li B, Yu H, et al (2013) An emerging hemorrhagic fever in China caused by a novel bunyavirus SFTSV. Sci China Life Sci 56: 697–700. 23917841 10.1007/s11427-013-4518-9

[10] Deng B, Zhou B, Zhang S, Zhu Y, Han L, et al (2013) Clinical features and factors associated with severity and fatality among patients with severe fever with thrombocytopenia syndrome Bunyavirus infection in Northeast China. PLoS One 8: e80802. 24236203 10.1371/journal.pone.0080802 PMC3827460

[11] Sun L, Hu Y, Niyonsaba A, Tong Q, Lu L, et al (2013) Detection and evaluation of immunofunction of patients with severe fever with thrombocytopenia syndrome. Clin Exp Med 10.1007/s10238-013-0259-0 PMC7101760 24068614

[12] Sun Y, Jin C, Zhan F, Wang X, Liang M, et al (2012) Host cytokine storm is associated with disease severity of severe fever with thrombocytopenia syndrome. J Infect Dis 206: 1085–1094. 22904342 10.1093/infdis/jis452

[13] Deng B, Zhang S, Geng Y, Zhang Y, Wang Y, et al (2012) Cytokine and chemokine levels in patients with severe fever with thrombocytopenia syndrome virus. PLoS One 7: e41365. 22911786 10.1371/journal.pone.0041365 PMC3404083

[14] Weng Y, Chen N, Han Y, Xing Y, Li J (2013) Clinical and laboratory characteristics of severe fever with thrombocytopenia syndrome in Chinese patients. Braz J Infect Dis 10.1016/j.bjid.2013.05.011 PMC9425216 24076112

[15] Ministry of Health, People's Republic of China (2010) National guideline for prevention and control of severe fever with thrombocytopenia syndrome. Available: http://www.moh.gov.cn/mohwsyjbgs/s8348/201010/49272.shtml Accessed 03 March 2014.

[16] Sun Y, Liang M, Qu J, Jin C, Zhang Q, et al (2012) Early diagnosis of novel SFTS bunyavirus infection by quantitative real-time RT-PCR assay. J Clin Virol 53: 48–53. 22024488 10.1016/j.jcv.2011.09.031

[17] Huang KJ, Su IJ, Theron M, Wu YC, Lai SK, et al (2005) An interferon-gamma-related cytokine storm in SARS patients. Journal Med Virol 75: 185–194. 15602737 10.1002/jmv.20255 PMC7166886

[18] Haque A, Hober D, Kasper LH (2007) Confronting potential influenza A (H5N1) pandemic with better vaccines. Emerg Infect Dis 13: 1512–1518. 18258000 10.3201/eid1310.061262 PMC2851514

[19] CDC (2009) Interim Guidance for Clinicians on Identifying and Caring for Patients with Swine-origin Influenza A (H1N1) Virus Infection. Centers for Disease Control and Prevention

